# Effect of Alumina Particles on the Osteogenic Ability of Osteoblasts

**DOI:** 10.3390/jfb13030105

**Published:** 2022-07-28

**Authors:** Ashish Ranjan Sharma, Yeon-Hee Lee, Buyankhishig Gankhuyag, Chiranjib Chakraborty, Sang-Soo Lee

**Affiliations:** 1 Institute for Skeletal Aging & Orthopedic Surgery, Hallym University-Chuncheon Sacred Heart Hospital, Chuncheon 24252, Korea; researchskeletal@gmail.com (A.R.S.); llyyhh2255@gmail.com (Y.-H.L.); buyankhishigtkh@gmail.com (B.G.); 2 Department of Biotechnology, School of Life Science and Biotechnology, Adamas University, Barasat-Barrackpore Rd, Kolkata 700126, India; drchiranjib@yahoo.com

**Keywords:** implant-induced osteolysis, alumina (Al_2_O_3_) particles, osteoprogenitors, WNT/β-catenin signaling pathway

## Abstract

Biomaterials are used as implants for bone and dental disabilities. However, wear particles from the implants cause osteolysis following total joint arthroplasty (TJA). Ceramic implants are considered safe and elicit a minimal response to cause periprosthetic osteolysis. However, few reports have highlighted the adverse effect of ceramic particles such as alumina (Al_2_O_3_) on various cell types. Hence, we aimed to investigate the effect of Al_2_O_3_ particles on osteoprogenitors. A comparative treatment of Al_2_O_3_, Ti, and UHMWPE particles to osteoprogenitors at a similar concentration of 200 μg/mL showed that only Al_2_O_3_ particles were able to suppress the early and late differentiation markers of osteoprogenitors, including collagen synthesis, alkaline phosphatase (ALP) activity and mRNA expression of Runx2, OSX, Col1α, and OCN. Al_2_O_3_ particles even induced inflammation and activated the NFkB signaling pathway in osteoprogenitors. Moreover, bone-forming signals such as the WNT/β-catenin signaling pathway were inhibited by the Al_2_O_3_ particles. Al_2_O_3_ particles were found to induce the mRNA expression of WNT/β-catenin signaling antagonists such as DKK2, WIF, and sFRP1 several times in osteoprogenitors. Taken together, this study highlights a mechanistic view of the effect of Al_2_O_3_ particles on osteoprogenitors and suggests therapeutic targets such as NFĸB and WNT signaling pathways for ceramic particle-induced osteolysis.

## 1. Introduction

Total joint arthroplasty (TJA) is a dramatically effective surgical therapy for the management of osteoarthritis and osteoporosis in hip and knee joints, reducing pain and improving mobility. TJA can resect the degenerative bone components and replace them with synthetic implants, recovering the worn joint surfaces [1]. Nowadays, the number of cases of TJA is drastically increasing due to the increased aging process and progressively active and long-lasting population. The cases of total hip arthroplasty (THA) are forecasted to increase by 673% and total knee arthroplasty (TKA) by 174% by 2030 in the United States of America [2]. Furthermore, in Korea, from 2006 to 2016, the TKA data showed an increase of 122%. An increasing proportion is of younger patients (<65 years), most of them being female [3]. A total of 20% of patients undergoing TJA required a joint revision post-operative surgery within 10 years [4]. Additionally, more frequent TJA revisions are expected as the implant durability period decreases more rapidly in highly active or younger people [5]. However, susceptibility toward periprosthetic osteolysis is often found to be associated with single nucleotide polymorphisms (SNPs) present in a particular human society [6]. Aseptic prosthetic loosening associated with osteolysis has become a typical cause of long-term failure of TJA [7,8,9]. Wear debris from the bearing interface is a significant factor in increasing the revision of surgeries and aggravating the implant’s life [10]. The wear debris can derive joint mechanical instability, decrease joint mobility, and increase joint pain with inflammatory biologic responses, leading to osteolysis and implant failure [10,11,12,13,14].

Usually, implant components are resistant to mechanical burden and enzymatic destruction, but, in the long run, continuously generated wear particles can interrupt the biological environment, causing the accumulation of phagocytes that devour the small particles of implants. Macrophages and other inflammatory cells can phagocyte the wear debris, causing cellular activation and secreting various pro-inflammatory cytokines (such as interleukin (IL)-1β, IL-6, IL-11, and TNF-α) [12,15,16,17]. These inflammatory cytokines can stimulate osteoclastogenesis, periprosthetic osteolysis, and, eventually, aseptic loosening. The macrophage lineage cells play a vital role in the differentiation of osteoclasts and wear-debris-induced implant loosening. Several researchers have shown that particular-sized wear debris initiates a composite pathology in periprosthetic osteolysis involving bone marrow stromal cells, osteoblasts, fibroblast cells, macrophages, monocytes, lymphocytes, and fibroblast-like synoviocytes. This often leads to an imbalance of osteoclastogenesis regulators such as RANKL and OPG [18,19,20,21,22]. Although bone loss by aseptic loosening of implants plays a critical role in bone resorption, the reduction in bone formation also appears to be a major factor contributing to bone resorption [23,24]. However, the role of implant-generated wear debris in affecting the osteogenic activity of osteoblasts has not been elucidated completely. It thus needs further research to understand their participation in wear debris-induced inflammation and impaired bone formation.

The osteoblast progenitor cells from bone marrow stromal cells (BMSCs) have an important role in maintaining bone homeostasis and are in close contact with the implant at the prosthetic site. The distress of osteoprogenitors arises from periprosthetic inflammation, and wear debris may reduce the differentiation and functions of the osteoblast cells, which can decrease osteogenesis. In addition, osteoprogenitors may play an essential role in the periprosthetic inflammation and wear debris associated with osteoclastogenesis [25]. The glycogen synthase kinase-3 (GSK-3) is a vital regulator in glycogen metabolism that regulates several biological processes of bone formation, involving bone remodeling during osteoblast cell differentiation and proliferation [26,27]. It has two major isoforms; among them, GSK-3β is more important for bone formation and degrades the β-catenin without WNT signals by forming a complex with APC and Axin [28]. It has been shown that β-catenin is a crucial factor for activating the WNT/β-catenin pathway, which has an essential role in bone formation. Suppressing this signaling pathway reduces the osteogenic process [29].

Nowadays, polyethylenes (such as ultra-high-molecular-weight polyethylene (UHMWPE)), ceramics (such as alumina (Al_2_O_3_)), and metals (such as titanium (Ti)) are the most-used essential biomaterials in TJA. Studies have indicated the effect of implant wear debris from metal implants (Ti, chromium (Cr), and cobalt (Co)), ceramic implants (Al_2_O_3_ and zirconia), and polymeric implants (UHMWPE and polymethylmethacrylate (PMMA)) during osteolysis [30,31]. The wear debris derived from these biomaterials shows numerous shapes (mostly spherical) and a profusely inflammatory or detrimental effect on osteoblast cells [32]. The researchers have indicated that the UHMWPE particle suppresses the osteoblast proliferation, viability, and differentiation of primary bone marrow osteoprogenitors in a dose-dependent manner [33]. Previously, it has been documented that differentiation of osteoprogenitors could inhibit PMMA particles in murine bone marrow cells [25,34]. Moreover, metal implant (Ti) particles may release pro-inflammatory factors from macrophage cells that stimulate osteoclastogenesis and suppress osteoblastogenesis [23]. However, the effects of particles from ceramic implant Al_2_O_3_ particles on osteoblast differentiation have not yet been fully elucidated and need detailed studies.

This study investigates the effect of Al_2_O_3_ particles on osteoprogenitors and the mechanism by which Al_2_O_3_ particles might regulate the process of bone loss during periprosthetic osteolysis.

## 2. Materials and Methods

### 2.1. Preparation of Al_2_O_3_, Ti, and UHMWPE Particles

The Al_2_O_3_ particles used in this study were procured from Sigma-Aldrich, USA (Cat. No. 544833). According to the manufacturer datasheet, the Al_2_O_3_ particles have the following characteristics: Appearance (Color): White; Appearance (Form): Powder; Size: ≤50 nm (TEM); Surface area: >40 m^2^/g (BET). Moreover, we also characterized the Al_2_O_3_ particles demonstrating multiple morphologies, including circular, oval, hexagonal, and spindle-shaped (Figure S1). The Al_2_O_3_ particles were suspended in PBS (100 mg/mL) and then autoclaved at 121 °C for 20 min. Pure Ti was purchased from the Johnson Matthey Company (UK) as a commercial product. Ti particles less than 10 μm in size were autoclaved at 180°C for 6 h. Then, they were washed for 48 h using 70% ethanol. UHMWPE (40–48 μm) was purchased from Sigma-Aldrich, St. Louis, MO, USA. The UHMWPE particles were washed and soaked for 48 h using 70% ethanol. All particles were suspended using phosphate-buffered saline (PBS), then refrigerated as stock solutions (100 mg/mL) at 4 °C. For the in vitro experiments, the particles were sonicated for 20 min before treatment to cells.

### 2.2. Cell Culture

MC3T3-E1 cell line (CRL-2593, ATCC, Manassas, VA, USA) and mice osteoblast precursor cell line were grown in α-minimum essential medium (α-MEM; Invitrogen, Grand Island, NY, USA) containing 10% fetal bovine serum (FBS; Gibco, Waltham, MA, USA), and 100 U/mL penicillin, 100 U/mL streptomycins (Invitrogen, Waltham, MA, USA), and 2 mM L-glutamine were added to α-MEM. Cells were maintained in a humidified incubator at 37 °C and 5% CO_2_.

### 2.3. MTT Assay

To evaluate the cell viability, MC3T3-E1 cells were stimulated with Al_2_O_3_, Ti, and UHMWPE particles at various doses (0, 25, 50, 100, 200, 400, 800, and 1000 μg/mL) for 24 h, and then 5 mg/mL of 3-(4,5-dimethylthiazol-2-yl)-2,5-diphenyl-tetrazolium bromide was added (MTT; Sigma Aldrich, St. Louis, MO, USA). The supernatant was removed after incubating with MTT at 37 °C for 2 h. Next, 200 μL of DMSO was added to each well, and we measured the optical density at 570 nm using a UV-Vis spectrophotometer (SpectraMax, Molecular Devices, San Jose, CA, USA).

### 2.4. Lactate Dehydrogenase Activity (LDH) Assay

The LDH released into the cell culture media was confirmed by a cytotoxicity detection kit (Roche Diagnostics, San Jose, CA, USA). MC3T3-E1 cells were treated with Al_2_O_3_, Ti, and UHMWPE particles at various doses (0, 25, 50, 100, 200, 400, 800, and 1000 μg/mL) for 24 h. Then, 10 μL cell-cultured media was transferred to a new 96-well plate. Subsequently, 50 μL LDH reagent and 40 μL of PBS were added to each well. After incubating for 45 min in the dark, we added 50 μL of stop solution to each well for stopping the enzymatic reaction. The optical density was measured using a UV-Vis spectrophotometer at 490 nm.

### 2.5. Alkaline Phosphatase (ALP) Activity

The ALP activity was analyzed as a marker of osteoblast differentiation. To analyze the ALP activity, MC3T3-E1 cells were stimulated with Al_2_O_3_, Ti, and UHMWPE particles at various doses (0, 25, 50, 100, 200, 400, 800, and 1000 μg/mL) for 24 h. A total of 60 μL of RIPA buffer was added to each well after washing the cells with cold PBS. The cell lysate from each well was centrifuged at 12,000 rpm for 20 min at 4 °C. Next, 20 μL of supernatant was transferred to a new tube and mixed with 100 μL of CSPD substrate, respectively. After incubating in the dark for 30 min, the intensity of luminescence was evaluated by a luminometer (Glomax, Promega, Madison, WI, USA). The protein concentration was normalized by using the protein assay kit.

### 2.6. Protein Isolation and Western Blotting

MC3T3-E1 cells were treated with 0, 100, 200, and 400μg/mL of Al_2_O_3_ particles for 24 h. The cell lysates were loaded into gels and transferred to the membrane. Then, 5% skim milk was used to block the membrane. After 1h, the blocked membrane was incubated with antibodies against Runx2, Col1α, Cox2, Iκbα, pSer9-GSK-3β, GSK-3β, β-catenin, and β-actin at 4 °C overnight. Next, it was washed using 1X TBST (Tris-buffered saline with 0.1% Tween 20) 3 times every 10 min. Afterward, the membrane was reacted with a horseradish peroxidase-conjugated secondary antibody at room temperature for 45 min. Chemiluminescence reagents were used to visualize target protein bands. β-actin was used as a loading control. The intensity of the band was quantified by Image J software (Version 1.53s, NIH, Bethsda, MD, USA).

### 2.7. RNA Isolation and Real-Time RT-PCR

MC3T3-E1 cells were stimulated with 200 μg/mL of Al_2_O_3_ particles. After incubating for 24 h, Trizol reagent (Invitrogen) was added to the cells to collect the total RNA. The purity and quality of the total RNA samples were determined carefully using the cuvette method. A total of 2 μg of RNA was used with SuperScript II (Invitrogen) for synthesized first-strand cDNA. 1 μL of cDNA, targeted primers, and EXPRESS SYBR green qPCR Supermix (Bioneer, Daejeon, Korea) are contained in each PCR blend. The real-time PCR analysis was performed as follows: the thermal cycle reaction at 95 °C for 10 min and the amplification of 40 cycles at 95 °C for 20 s, 60 °C for 20 s, and 72 °C for 25 s. Glyceraldehyde 3-phosphate dehydrogenase (GAPDH) was used to normalize each sample. Quantification was measured by ∆∆CT method. The primer sequences are recorded in Table 1.

### 2.8. Luciferase Reporter Assay

MC3T3-E1 cells were treated with 200 µg/mL of Al_2_O_3_ particles for 24 h after transfecting reporter constructs using Genefectine reagent (Genetrone Biotech, Jeonju-si, Korea) according to the manufacturer’s protocol. Luciferase activity was estimated by treating with 1 µg of Axin-2 and the Renilla luciferase thymidine kinase constructs in the cells. Luciferase activities were evaluated using a luminometer. Normalization of Axin-2 reporter activity from each sample was evaluated using Renilla luciferase activity.

### 2.9. Sirius Red S Staining

MC3T3-E1 cells were treated with Al_2_O_3_ particles for 5 days in a 48-well plate. Cells were fixed with Bruin’s fluid (Sigma Aldrich, St. Louis, MO, USA) at room temperature for 1 h and were washed with distilled water 5–7 times. Next, cells were stained with Sirius Red S solution (Sigma Aldrich, St. Louis, MO, USA) for 1 h. Stained cells were dissolved in 0.1 N sodium hydroxide for 30 min and then were detected using a spectrophotometer at 550 nm. Additionally, blank was detected by adding 0.1 N sodium hydroxide to the empty well.

### 2.10. Statistical Analysis

All data were analyzed statistically using Prism 5.0 software (Graphpad, San Diego, CA, USA) and were evaluated by the *p*-value of a two-tailed Student’s *t*-test (* *p* < 0.05 and ** *p* < 0.01).

## 3. Results

### 3.1. Comparison of Cell Morphology, Viability, and Cytotoxicity after Stimulating Osteoprogenitors with Different Types of Wear Particles

**Figure 1:**
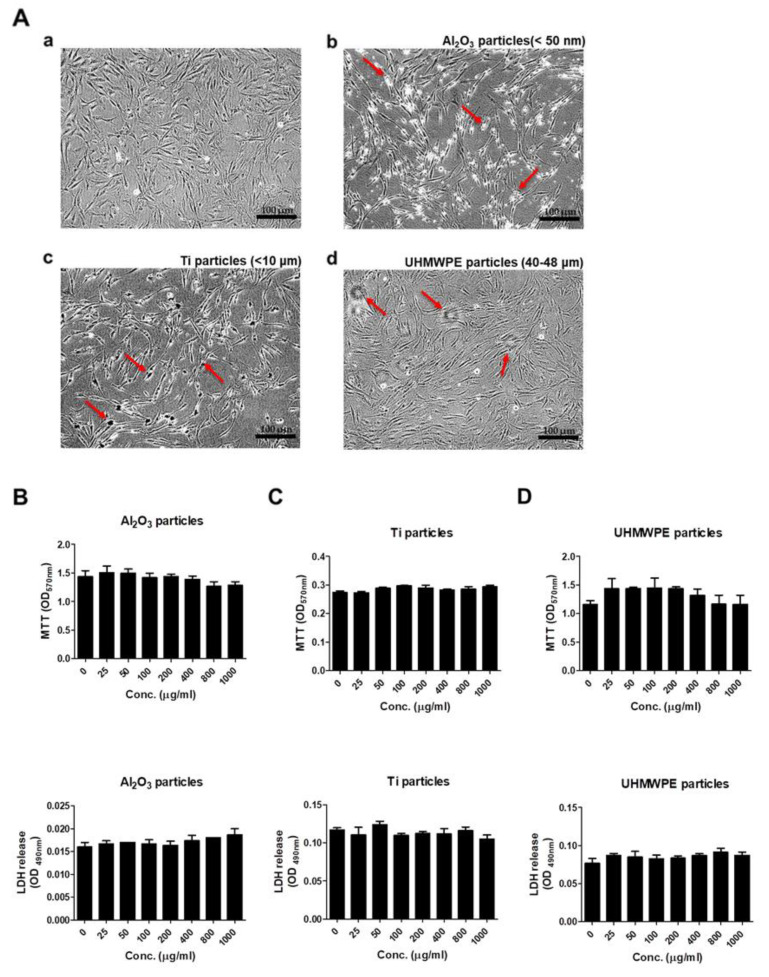
Comparison of cell morphology, viability, and cytotoxicity after stimulating osteoprogenitors with Al_2_O_3_, Ti, and UHMWPE particles. (**A**) The microscope pictures showed a change in cell morphology after stimulation by (**b**) Al_2_O_3_ particles, (**c**) Ti particles, and (**d**) UHMWPE particles on MC3T3-E1 cells for 48 h, respectively. (**b**) Al_2_O_3_ particle-, (**c**) Ti particle- or (**d**) UHMWPE particle-treated cells showed a difference in morphology compared to the control (**a**). Arrows indicate Al_2_O_3_, Ti or UHMWPE particles treated to the cells. Magnification ×10. Cell viability and cytotoxicity were evaluated after stimulating various concentrations of (**B**) Al_2_O_3_ particles, (**C**) Ti particles, or (**D**) UHMWPE particles on MC3T3-E1 cells for 24 h, respectively. All three particles had no effect on the MC3T3-E1 cells. Similar results were obtained in three independent experiments.

To assess any effect of Al_2_O_3_, Ti, and UHMWPE particles on the morphology of MC3T3-E1 cells, cells were treated for 48 h, and changes in morphology were observed under a microscope. Treatment of equal concentrations of Al_2_O_3_, Ti, and UHMWPE particles (200 μg/mL) altered the morphology of MC3T3-E1 cells compared to the control (Figure 1A(a–d)). To observe any effect of various concentrations of Al_2_O_3_, Ti, and UHMWPE particles on the viability and cytotoxicity of MC3T3-E1 cells, cells were treated with particles for 24 h, and MTT and LDH assays were performed. No changes in the viability or cytotoxicity of MC3T3-E1 cells were observed after the treatment of Al_2_O_3_, Ti, and UHMWPE particles for 24 h (Figure 1B–D).

### 3.2. Al_2_O_3_ Particles Suppress ALP Activity of Osteoprogenitors

**Figure 2:**
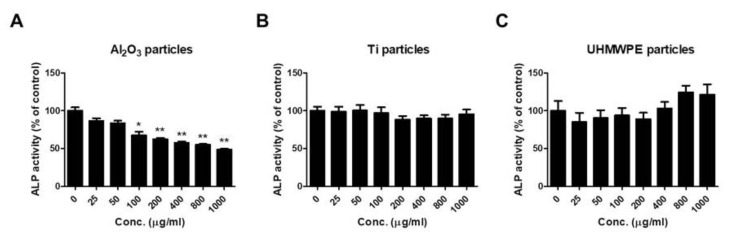
Al_2_O_3_ particles suppress the ALP activity of osteoprogenitors. MC3T3-E1 cells were treated with the wear particles (Al_2_O_3_, Ti, and UHMWPE particles) for 48 h. While ALP activity was dose-dependently notably decreased in (**A**) Al_2_O_3_ particle-treated MC3T3-E1 cells, no significant changes in (**B**) Ti- or (**C**) UHMWPE particle-treated MC3T3-E1 cells were observed. Data are shown as the mean ± SD. Three independent experiments were performed to obtain similar results. * *p* < 0.05, ** *p* < 0.01 compared to the control.

To confirm any effect of Al_2_O_3_, Ti, and UHMWPE particles on ALP activity, MC3T3-E1 cells were treated with the respective particles at various doses. After 24 h of treatment, the ALP activity of the cells was determined (Figure 2). Treatment of Al_2_O_3_ particles dose-dependently reduced the ALP activity of MC3T3-E1 cells (Figure 2A). In contrast, Ti and UHMWPE particles showed no changes in the ALP activity of MC3T3-E1 cells (Figure 2B,C).

### 3.3. Effects of Al_2_O_3_ Particles on the Osteogenic Activity of Osteoprogenitors

**Figure 3:**
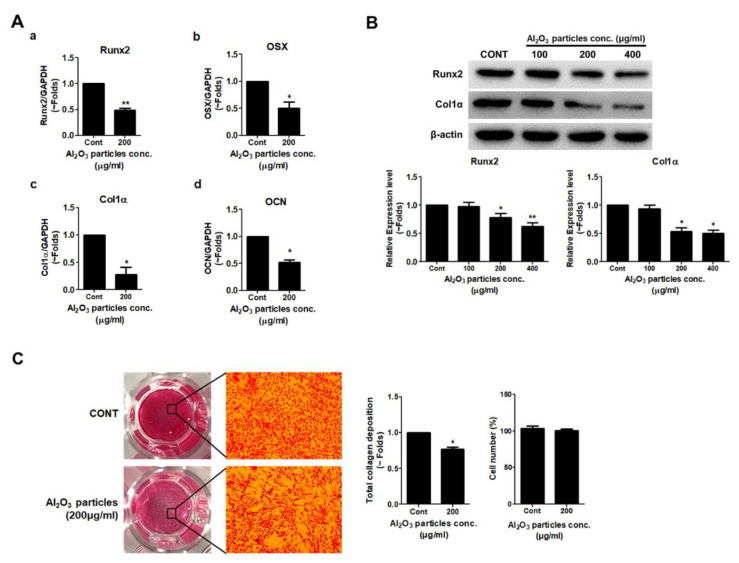
Al_2_O_3_ particles inhibit the expression of osteogenic differentiation markers. MC3T3-E1 cells were stimulated with Al_2_O_3_ particles (200 μg/mL) for 24 h. (**A**) mRNA expression levels of (**a**) Runx2, (**b**) OSX, (**c**) Col1α, and (**d**) OCN were analyzed by RT-PCR. (**B**) Protein expression levels of Runx2 and Col1α were detected by Western blotting. To assess the effect of Al_2_O_3_ particles on collagen synthesis in osteoprogenitors, MC3T3-E1 cells were treated with Al_2_O_3_ particles (200 μg/mL) for 5 days. (**C**) Collagen synthesis was detected by Sirius Red S staining. Data are shown as the mean ± SD. Three independent experiments were performed to obtain similar results * *p* < 0.05, ** *p* < 0.01 compared to the control.

Since Al_2_O_3_ particles significantly suppressed the ALP activity of MC3T3-E1 cells dose-dependently, an attempt was made to verify the effect of Al_2_O_3_ particles on other osteogenic parameters. Since a dose of 200 μg/mL of Al_2_O_3_ particles showed no effect on the viability and cytotoxicity of MC3T3-E1 cells and even suppressed the ALP activity significantly, a dose of 200 μg/mL was utilized for further experiments. RT-PCR results showed that Al_2_O_3_ particles remarkably reduced the mRNA expression of Runx2, OSX, Col1α, and OCN in comparison to control (Figure 3A). MC3T3-E1 cells were treated with Al_2_O_3_ particles for 24 h, and the protein was collected. Western blot results demonstrated that the treatment of Al_2_O_3_ particles significantly reduced the protein expression of osteoblastic differentiation markers, Col1α, and osteoblastic transcription factor, Runx2, in MC3T3-E1 cells (Figure 3B). The effect of Al_2_O_3_ particles on the collagen synthesis of MC3T3-E1 cells was evaluated with Sirius Red S staining, and quantification was performed using a colorimetric assay after 5 days of treatment (Figure 3C). The results demonstrate that collagen synthesis was significantly inhibited in Al_2_O_3_ particle-treated cells compared to the control.

### 3.4. Al_2_O_3_ Particles Induce Inflammation in Osteoprogenitors

**Figure 4:**
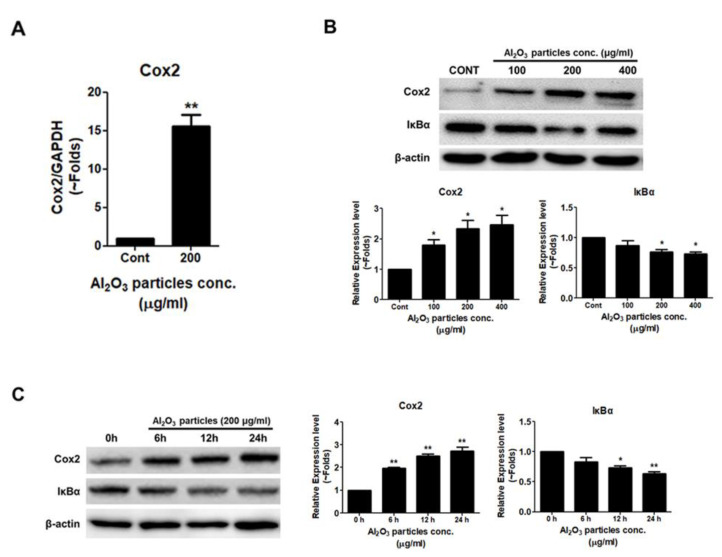
Al_2_O_3_ particles induce inflammation in osteoprogenitors. (**A**) MC3T3-E1 cells were treated with Al_2_O_3_ particles (200 μg/mL) for 24 h. RT-PCR was used to analyze the mRNA expression level of Cox2. (**B**) Al_2_O_3_ particles were treated dose-dependently (100, 200, and 400 µg/mL) to MC3T3-E1 cells for 24 h, and (**C**) time-dependently (6, 12, and 24 h) with 200 µg/mL of Al_2_O_3_ particles. Cox2 and Iκbα protein expression levels were analyzed by Western blotting. Data are shown as the mean ± SD. Three independent experiments were performed to obtain similar results * *p* < 0.05, ** *p* < 0.01 compared to the control.

RT-PCR analysis showed that after treatment with Al_2_O_3_ particles of MC3T3-E1 cells at 24 h, the mRNA expression level of inflammatory marker Cox2 was increased (Figure 4A). Even Western blot analysis showed a dose-dependent and time-dependent increase in Cox2 expression after 24 h of Al_2_O_3_ particle treatment to MC3T3-E1 cells (Figure 4B,C). Moreover, the stability of Iκbα was decreased after the treatment with Al_2_O_3_ particles of MC3T3-E1 cells in a dose-dependent and time-dependent manner (Figure 4B,C), implicating the induction of NFĸB signaling activity.

### 3.5. Al_2_O_3_ Particles Reduce the Activity of the WNT/β-Catenin Signal Pathway

**Figure 5:**
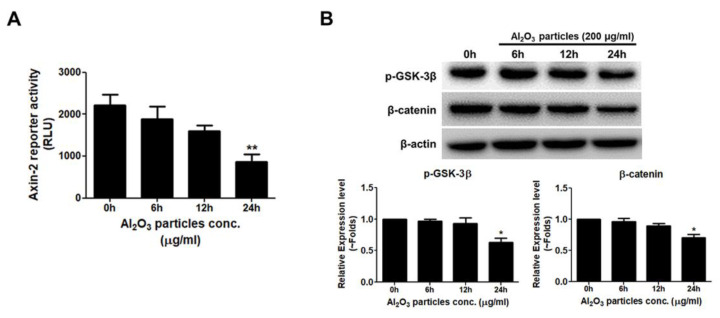
Al_2_O_3_ particles reduce the osteogenic activity through WNT/β-catenin signaling pathway. (**A**) The Axin-2 reporter construct was transfected to MC3T3-E1 cells, and a luciferase assay was performed. The Axin-2 activity was reduced time-dependently in Al_2_O_3_ particles-treated cells. (**B**) Protein expression levels of pSer9-GSK-3β and stabilization of β-catenin were determined by Western blotting, and both decreased in Al_2_O_3_ particles-treated cells. Data are shown as the mean ± SD. Three independent experiments were performed to obtain similar results * *p* < 0.05, ** *p* < 0.01 compared to the control.

Next, any effect on the WNT/β-catenin signaling pathway activation by Al_2_O_3_ particles was analyzed in MC3T3-E1 cells. The WNT/β-catenin signaling pathway is well-known to play an important role in regulating the differentiation of osteoblast and bone formation [35]. MC3T3-E1 cells were transfected with Axin-2 luciferase reporter construct for 24 h using FuGENE 6 and treated with Al_2_O_3_ particles, and the effect was analyzed after 6, 12, and 24 h. Al_2_O_3_ particles significantly decreased Axin-2 luciferase activity in a time-dependent manner after 24 h (Figure 5A). Western blotting protein quantification after 0, 6, 12, and 24 h of treatment of Al_2_O_3_ particles on MC3T3-E1 cells showed decreased expression levels of pSer9-GSK-3β. Similarly, a decreased stability of β-catenin was also observed in Al_2_O_3_ particles treated with MC3T3-E1 cells (Figure 5B).

### 3.6. Al_2_O_3_ Particles Increase the mRNA Expression Level of the WNT/β-Catenin Signaling Pathway Antagonists

**Figure 6:**
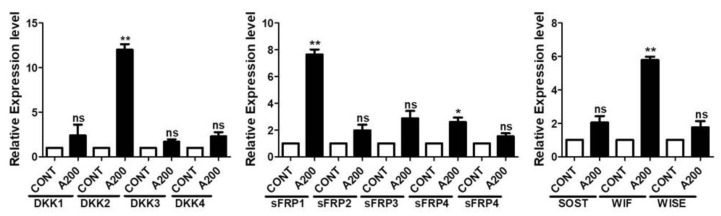
Al_2_O_3_ particles increase the mRNA expression level of the WNT signaling antagonists. MC3T3-E1 cells were treated with Al_2_O_3_ particles (200 μg/mL) for 24 h. RT-PCR was used to analyze the mRNA expression level of WNT signaling antagonists. Data are shown as the mean ± SD. Three independent experiments were performed to obtain similar results * *p* < 0.05, ** *p* < 0.01, ns (not significant) compared to the control.

The WNT/β-catenin signaling pathway is under the endogenous control of various antagonists. A number of antagonists control the WNT/β-catenin signaling pathway at various stages of its activation and regulate its participation in the physiological outcome [36]. Thus, mRNA expression levels of various WNT/β-catenin signaling pathway antagonists were screened after treatment with Al_2_O_3_ particles for the MC3T3-E1 cells (Figure S1). The mRNA expression levels of several antagonists of the WNT/β-catenin signaling pathway increased, such as the DKK family, sFRP family, and WIF. Notably, the expression of DKK2 (~12 fold), sFRP1 (~7 fold), and WIF (~6 fold) were found to be significantly higher (Figure 6).

## 4. Discussion

TJA cases have been increasing recently, yet aseptic loosening is the major cause of concern for implant failure and infection problems. Implant wear from prosthesis is unavoidable due to articulation at the surfaces of the prosthesis. Wear debris generated from the prosthesis usually leads to chronic inflammation and ultimately to bone resorption. Moreover, wear debris also causes mechanical instability, reduces mobility, and increases joint pain. These factors often lead to osteolysis and finally cause loosening and failure of the implants [14]. Several new materials have been developed for TJA, such as Ti-based alloy, cobalt-chromium-based alloy (CoCr), carbon fiber-reinforced (CFR), carbon-carbon composite, polycarbonate-urethane (PCU), cross-linked polyethylene, and ceramic-ceramic composite, along with engineered surfaces to minimize the risk of osteolysis. These newly developed materials are more inert and have a minimal wear rate to avoid periprosthetic osteolysis. However, increasing revision cases post-TJA are still a concern and need further studies to improve the implant materials and biocompatibility. How wear debris from prostheses can lead to the progression of aseptic loosening depends on the characteristics of the debris.

The sizes, shapes, and chemical compositions of implant debris and the biological response to them by various cell types define the subsequent complications. Usually, the wear debris from the prosthesis ranges from the micron to sub-micron range. However, the majority of them are less than 5 mm in diameter [37]. Due to increasing free radical production and inducing chromosomal damage, nano-sized wear debris is potentially the most harmful range [38]. However, different kinds of wear debris may have different size ranges to elicit biological responses. For example, for UHMWPE wear debris, the size and volumetric concentration are crucial factors for any tissue response. UHMWPE particles in the size range of 0.2–7 µm have been found to be most biologically reactive. UHMWPE in a size range of (average diameter) 6.54 μm ± 4.43 μm has been shown to inhibit the osteogenesis potential of fibroblasts through the activation of macrophages [39]. Moreover, osteoblasts challenged with UHMWPE particles show a much higher RANKL/OPG ratio compared to the control [40]. Ti particles (<10 μm) have been shown to affect the osteogenic activity of osteoblasts not only when in direct contact, but also even through macrophages [41]. Due to more extended durability, chemical inertness, resistance to corrosion, non-allergic properties, low wearing rate, and reduced risk of osteolysis, ceramic-on-ceramic implants are favorable for TJA [42,43]. However, ceramic implants are also associated with limitations such as slow crack growth, brittleness, and sound associated with implants, namely squeaking, clicking, and popping [44,45,46]. Even during the course of their use, histological studies have revealed that ceramic implants do generate wear particles, but not-so-serious foreign body granulomas are observed around the prosthesis compared to metal implants [47].

Nevertheless, one of the constituents of ceramics, Al_2_O_3_ particles (<50 nm), has been shown to regulate negative feedback of RANKL expression in fibroblasts and reduce osteolysis [48]. Likewise, another study also observed no significant effect of Al_2_O_3_ particles on osteoblast-like cells and macrophage-like cells [49]. Inversely, the co-culturing of osteoblasts and macrophages in the presence of Al_2_O_3_ particles initiated an inflammatory response by releasing inflammatory mediators such as cytokines (IL-6 and TNF-α) [50]. Considering the contrary and not-so-apparent effect of Al_2_O_3_ in osteolysis during implant-induced bone loss, the current study aimed to evaluate the effect of Al_2_O_3_ and the mechanism underlying the effect, if any, on the osteogenic activity of the osteoprogenitor. Moreover, a comparison of the effect of UHMWPE, Ti, and Al_2_O_3_ particles on the osteogenic activity of osteoprogenitors was also evaluated.

Treatment of UHMWPE (40–48 µm), Ti (<10 µm), and Al_2_O_3_ (<50 nm) particles on MC3T3-E1 cells at a dose of 200 μg/mL demonstrated a change in the morphology of MC3T3-E1 cells compared to the control (Figure 1A). A change in morphology may be due to the ability of osteoblasts to phagocytose wear debris of particular sizes [51]. Since a clinically relevant size of the debris was taken for the study, a change in the cells’ morphology may be due to the phenomenon of internalization, specifically phagocytosis. Next, to address any effect of these particles on the viability or cytotoxicity of the osteoprogenitor, MC3T3-E1 cells were treated with Al_2_O_3_, Ti, and UHMWPE particles at several doses (0, 25, 50, 100, 200, 400, 800, and 1000 μg/mL) and effects on the cytotoxicity and cell viability of MC3T3-E1 cells were evaluated. Results demonstrated that none of the wear particles had a significant effect on the cytotoxicity and cell viability of MC3T3-E1 cells (Figure 1B–D). ALP is regarded as an early differentiation marker for osteoblasts. Treatment with various doses of the Al_2_O_3_, Ti, and UHMWPE particles on MC3T3-E1 cells showed that except for Al_2_O_3_ particles, no other particles (UHMWPE and Ti) had any effect on the ALP activity of MC3T3-E1 cells (Figure 2). Dose-dependently, Al_2_O_3_ particles decreased the ALP activity (Figure 2A). Previous studies have shown that alumina wear debris in a size range of 2 nm to 90 nm (as measured by TEM) and 0.05–3.2 µm (as measured by SEM) can be generated from ceramic-on-ceramic joints [52]. However, nano-sized ceramic particles are found to be more bioactive than nano-sized particles of other materials [49]. Moreover, it has been reported that a larger volume of alumina particles (nm size range) is required to elicit biological response compared to the µm size range [53]. Thus, in our case, alumina particles of size ≤ 50 nm at a dose of 200 μg/mL were able to induce a suppressive effect on the ALP activity of osteoprogenitors.

Since ALP activity represents the early differentiation process of osteoprogenitors to osteoblasts, any effect of Al_2_O_3_ particles on other parameters of osteogenesis needed to be defined. Al_2_O_3_ particles upon treatment to MC3T3-E1 cells decreased the expression of osteogenic transcriptional factors such as Runx2 and OSX and osteogenic markers such as Col1α and OCN (Figure 3). Since Runx2 acts as the master regulator of the osteoblast differentiation process and Col1α is regarded as a marker for collagen synthesis for bone formation, protein expression of these proteins was confirmed [54]. In a dose-dependent manner, Al_2_O_3_ particles suppressed the expression of Runx2 and Col1α (Figure 3A). Moreover, a suppressed collagen synthesis of the MC3T3-E1 cells was observed after the treatment of Al_2_O_3_ particles (Figure 3C). These data confirm and validate the anti-osteogenic activity of Al_2_O_3_ particles on MC3T3-E1 cells.

Previous studies have demonstrated that wear debris induces an inflammatory response in cells near the prosthesis and is a cause of concern for bone loss during periprosthetic osteolysis [55]. Induced expression of Cox2 along with NFĸB signaling pathway activation confirmed an inflammatory response of MC3T3-E1 cells to Al_2_O_3_ particles (Figure 4). Al_2_O_3_ particles increased the Cox2 expression and activity of the NFĸB signaling pathway in a dose- and time-dependent manner, implicating the inflammatory state of MC3T3-E1 cells in response to Al_2_O_3_ particles.

Suppression of the expression of Runx-2, OSX, and other osteogenic markers in Al_2_O_3_ particles treating MC3T3-E1 cells required the assessment of the Al_2_O_3_ on key osteogenic signaling, such as WNT and BMP signaling pathways. Al_2_O_3_ significantly suppressed the WNT signaling activity, whereas no effect on the BMP signaling was observed (data not shown). The WNT/β-catenin signaling pathway is crucial for cell multiplication, differentiation, and cell survival of the osteogenic lineage during skeletal development [56]. After treatment of Al_2_O_3_ particles on MC3T3-E1 cells, the suppression of WNT/β-catenin signaling activity by Al_2_O_3_ particles explains the decrease in the osteogenic activity of osteoprogenitors in our system (Figure 5). Moreover, significantly increased expression of WNT/β-catenin signaling antagonists (DKK2, sFRP1, and WIF) in Al_2_O_3_ particle-treated MC3T3-E1 cells explain the downregulation of WNT/β-catenin signaling activity and thus the osteogenic activity of osteoprogenitors (Figure 6). Thus, NFĸB and WNT/β-catenin signaling pathways might be targeted for the treatment of ceramic-induced osteolysis.

## 5. Conclusions

This study highlights the anti-osteogenic characteristics of Al_2_O_3_ particles on osteoprogenitors. Ceramic implants are considered safe and are known to elicit a minimal response to cause periprosthetic osteolysis. Nevertheless, once the wear debris is generated from the articulated ceramic surfaces, wear debris such as Al_2_O_3_ particles can induce an inflammatory response in osteoblasts and suppress osteogenic activity to cause bone loss. An assessment of wear debris from periprosthetic tissues revealed that particles in the nm size range are more biologically active than the larger size (µm). In corroboration, our results have shown that Al_2_O_3_ particles in nm size have anti-osteogenic activity compared to the other wear debris analyzed in this study. Thus, clinicians and future researchers should carefully analyze the amount and size of wear debris generated from the ceramic implants of joint replacement surgeries and take necessary clinical interventions to avoid trauma associated with the failure of implants in long-term use. Moreover, further studies are required to analyze the effect of Al_2_O_3_ particles even on other cells such as macrophages, synoviocytes, osteocytes, and other cell types that are known to participate in implant-induced osteolysis. The limitation of our study is that we have investigated the effect of Al_2_O_3_ particles only on the murine osteoprogenitor cell line, MC3T3 E-1 cells; thus, further studies would be required to validate our results in more relevant in vitro osteolysis models comprising human primary osteoblasts, MSCs or in other in vivo osteolysis model.

Taken together, this study highlights a mechanistic view of the effect of Al_2_O_3_ particles on osteoprogenitors and suggests therapeutic targets such as NFĸB and WNT signaling pathways for ceramic particle-induced osteolysis.

## Figures and Tables

**Table 1:** Real-time RT PCR primer.

Gene	Primer Sequence (5′-3′)
Cox2	F: AAGTGCGGTGCAAACTTTCT R: TCTCGGTGGCTGGTAGTGA
Runx2	F: GGAAAGGAGGCACAAAGAAGCCAT R: AGTCCATTGGTGCTTGAGAAGGGA
Osterix (OSX)	F: TTCTCCTGGCAAAGACGGAC R: AGGAAGCTGAAGTCATAACCGCCA
Collagen 1α (Col1α)	F: TGCTTGTGACGAGCTATCAG R: GAGGACAGGGAGGATCAAGT
Osteocalcin (OCN)	F: TTCAAAAGAAGTGCTGGAAAAGGT R: GATCATCTCTACCTGAGTGTCTTT
DKK1	F: TCAGGTCCATTCTGGCCAACTCTT R: TGGGCATTCCCTCCCTTCCAATAA
DKK2	F: ATGGCAGAATCTAGGAAGGCCACA R: CGAACCCTTCTTGCGTTGTTTGGT
DKK3	F: AGCTGATGGAAGACACTCAGCACA R: TCCTGGTGCACATGGACTGTGTTA
DKK4	F: ATGGTACTGGTGACCTTGCTTGGA R: TCCGCGGAGCTCTTGATGTTGTTA
sFRP1	F: ACGAGTTGAAGTCAGAGGCCATC R: ACAGTCGGCACCGTTCTTCAG
sFRP2	F: ATCCTGGAGACAAAGAGCAAGACC R: TGACCAGATACCGGAGCGTTGATG
sFRP3	F: TGCAAATGTAAGCCTGTCAGAGC R: TCCACAACGGCGGTCACATC
sFRP4	F: GTGGCGTTCAAGGATGATGCTTC R: TTACTGCGACTGGTGCGACTG
sFRP5	F: CCCTGGACAACGACCTCTGC R: CACAAAGTCACTGGAGCACATCTG
Sclerostin (SOST)	F: AAAGGGAAGGGAGTGTGGAACGAA R: CGCAGGCTTTACATTTGGGTGGAA
WIF	F: CCACCTGAGGAGAGCTTGTACC R: TGGCATTCTTTGTTGGGCTTTCC
WISE	F: ACTGGATCGGAGGAGGCTATGG R: TGTGGCTGGACTCGTTGTGC
GAPDH	F: TCGTGGATCTGACGTGCCGCCTG R: CACCACCCTGTTGCTGTAGCCGTAT

## Data Availability

Not applicable.

## Supplementary Materials

The following supporting information can be downloaded at: https://www.mdpi.com/article/10.3390/jfb13030105/s1, Figure S1: Characterization of Al_2_O_3_ particles.
