# Supplementary material for: Effect of Alumina Particles on the Osteogenic Ability of Osteoblasts
Source: J Funct Biomater. 2022 Jul 28;13(3):105. doi: 10.3390/jfb13030105 (PMC9397023; doi:10.3390/jfb13030105)
Supplement: Supplementary file 1 [file jfb-13-00105-s001.zip › jfb-1828391-supplementary.pdf]

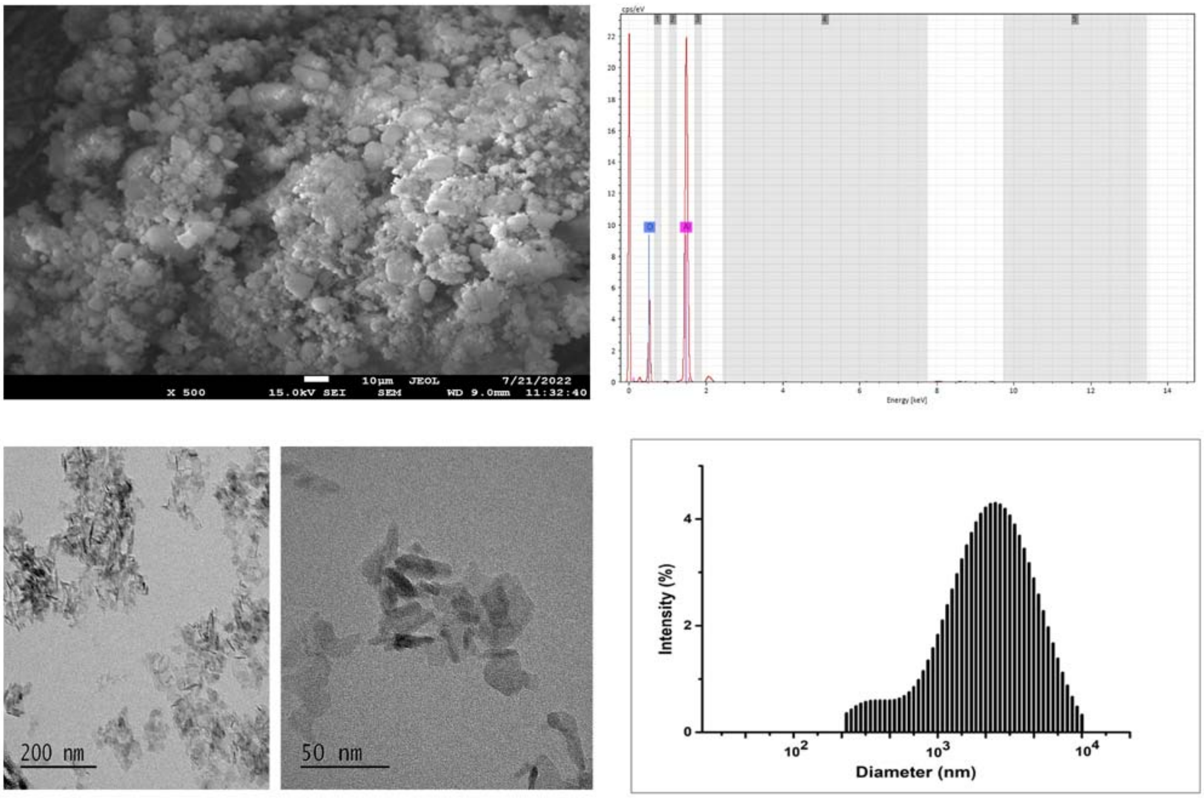

Figure S1. **Characterization of Al_2O_3 particles.**

The morphology of Al_2O_3 particles was observed using TEM (JEOL, JEM-2100F, Tokyo, Japan) and SEM/EDS (JEOL, JSM-7001F, Tokyo, Japan). For TEM, the dispersion of Al_2O_3 particles was loaded on the formvar-coated copper grid and air dried. For SEM, powdered samples were loaded on a platinum grid and pretreated with Ar plasma at 10 mA for 60 sec in a Cressington sputter coater. The hydrodynamic size distribution of Al_2O_3 particles was observed using a DLS spectrophotometer (Zetaplus 90, Brookhaven Instrument Co., USA).

The TEM images revealed clusters of Al_2O_3 particles (less than 50 nm) with multiple morphologies, including circular, oval, hexagonal, and spindle-shaped. The morphologies observed by TEM images are in accordance with SEM images which also show clusters of Al_2O_3 particles with multiple morphologies. The EDS mapping shows the presence of only aluminum and oxygen, confirming the purity of Al_2O_3 particles. However, the average hydrodynamic size of Al_2O_3 particles was observed as 1800.7 nm, and the polydispersity index (PDI) was 0.524. The hydrodynamic size of Al_2O_3 particles is more than the size of the Al_2O_3 particles as observed in TEM images, possibly due to the clusters of Al_2O_3 particles.
